# Communication of health messages using theatre: Appreciative inquiry from Ubuntu theatre group

**DOI:** 10.4102/hsag.v29i0.2622

**Published:** 2024-07-10

**Authors:** Gopolang Gause, Rachel T. Lebese, Fhumulani M. Mulaudzi, Molekodi J. Matsipane, Seepaneng S. Moloko-Phiri, Shonisani Tshivhase

**Affiliations:** 1 Lifestyle Diseases Research Focus Area, Faculty of Health Sciences, North-West University, Mahikeng, South Africa; 2 Department of Advanced Nursing Science, Faculty of Health Sciences, University of Venda, Thohoyandou, South Africa; 3 Department of Nursing Science, Faculty of Health Sciences, University of Pretoria, Pretoria, South Africa; 4 NuMIQ Research Focus Area, Faculty of Health Sciences, School of Nursing, North-West University, Mahikeng, South Africa; 5 Department of Public Health, Faculty of Health Sciences, University of Venda, Thohoyandou, South Africa

**Keywords:** appreciative inquiry, communication, health messages, innovation, Ubuntu, theatre

## Abstract

**Background:**

Theatre involves expressing meaning in a collaborative art using words, movements, and visual elements. However, theatre remains poorly used as a viable teaching strategy or a method for communicating health messages. Instead, it is relegated to solemnly transmitting indigenous knowledge.

**Aim:**

To explore and describe communication of educational health messages through theatre using an appreciative inquiry approach.

**Setting:**

The study was conducted among the Ubuntu theatre group from a rural province in South Africa. The group is famous for using theatre to communicate educational health messages through the lens of Ubuntu philosophy.

**Methods:**

A qualitative exploratory descriptive design was followed. A non-probability purposive sampling was used to select thirteen members of the Ubuntu theatre group. Data were collected by two moderators from the two focus group discussions through conference calls. Deductive thematic content data analysis was used to describe the 4-Ds of appreciative inquiry.

**Results:**

Theatre is a playful pedagogy that can cut through language and cultural barriers when used to communicate educational health messages. There is a need to formalise it as an alternative pedagogy within the health care sciences curriculum. Furthermore, the sustainability of theatre as an educational tool is dependent on expanding educational practices, documenting its success stories and periodical in-service training.

**Conclusion:**

Using Ubuntu innovation to communicate complex educational health messages through theatre can maximise learning. This study recommends that Ubuntu-infused health messages be conveyed using theatre.

**Contribution:**

The study adds to the body of knowledge by presenting Ubuntu innovation in communicating health messages through theatre.

## Introduction

Theatre involves expressing meaning in a collaborative art which is a combination of words, voices, movement and visual elements (Malloy 2022; Yi-Man 2002). The art form of theatre is often recognised as a creative and engaging method for educating individuals across various settings. From visual arts to music and creative drama, theatre offers a wide range of possibilities for fostering innovation in education (Excell & Van As 2018). While commonly associated with entertainment, theatre can also serve as a powerful means of conveying information and promoting health-related messages. Throughout history, Indigenous knowledge has been passed down across generations through the art of storytelling, song and dance. According to Keane, Khupe and Muza (2016), storytelling is an essential component of preserving Indigenous knowledge. In Africa, theatre has played a crucial role in maintaining intergenerational dialogue about Indigenous knowledge (Parker 2018). It brought the young and old together as part of collectivism and participatory decision-making as the principles of the philosophy of Ubuntu.

The Ubuntu philosophy within geospatial Africa is a dominant ontological system of humanness used to bring all cultures together through its principles (Mokhachane et al. 2023; Ngondo & Klyueva 2022). According to Muhammad-Lawal et al. (2023), the concept of Ubuntu is an African philosophy that underpins holistic care within communities. As a result, it is imperative to integrate the Ubuntu philosophy with the related African Indigenous knowledge systems within the health care system from as early as the training years (Moeta et al. 2019). The infusion of communication of multicultural messages has, for the longest time, played an integral part in curbing poor health practices while promoting health. For example, folktales enacted by knowledgeable indigenous individuals have been used to engage audiences and convey educational and health messages through interactive learning experiences that involve singing, rhythmic clapping and asking questions. Magwaza and Zuma (2020) emphasise that the audience actively participates in these performances, creating a shared experience that promotes cultural preservation and growth.

According to Sonke et al. (2018), communication of educational health messages is classified under primary health care as a form of prevention and promotion of health. Furthermore, the authors assert that the communication of educational health messages is intended to inform and educate communities about issues that affect their health. For example, a study conducted by Massar et al. (2018) in rural Zambia cited that theatre is commonly used to increase health awareness among communities, including maternal health. Its success in communicating educational health messages is attributed to its ability to demystify health issues and break communication barriers through creative arts (Eren & Can 2022; Mbizvo 2006). Furthermore, theatre is a preferred method to communicate educational health messages because of its track record of educating the public and ensuring community engagement to achieve social change (Rangiwhetu et al. 2020; Sonke et al. 2018). This embodies the principle of communalism as expressed in Ubuntu philosophy.

Ubuntu-centred communication approaches are highly preferred as they have the potential to converge individual and social perspectives of human behaviour towards a common goal of promoting the well-being of individuals or communities (Sonke et al. 2018). A study conducted in Kenya by Sonke et al. (2018) revealed that theatre has gained popularity in recent times. This is confirmed by a study conducted in Australia on the use of drama which identified that the use of drama as a method of teaching enabled students to acquire knowledge and life skills like empathy through shared content improvisation and the exploration of powerful, historically valuable, Indigenous theatre texts that reflect Indigenous perspectives (Williams & Morris 2022). Furthermore, the Ubuntu model in the nursing project in South Africa used theatre successfully as a vehicle for conveying educational health information to communities for 4 years (*Centering Ubuntu Healthcare in Society 5.0: A transdisciplinarity conference, South Africa, Pretoria, University of Pretoria*). Nevertheless, theatre remains poorly used as a teaching strategy in various teaching and learning institutions, except in drama schools. Instead, more than often, theatre is relegated to being used as a tool for transmitting Indigenous knowledge. Therefore, it is crucial to examine successful examples of using theatre to convey educational health messages through appreciative inquiry methods.

### Research aim

The study aims to explore and describe communication of educational health messages through theatre.

## Research design and methods

### Research design

**FIGURE 1:**
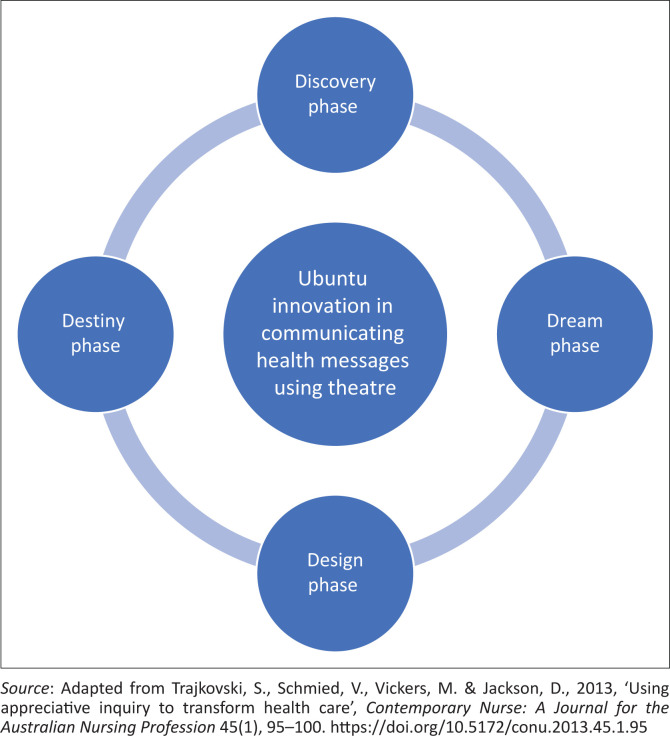
4-D relational cycle of appreciative inquiry.

A qualitative, exploratory and descriptive design was used to explore and describe communication of educational health messages through theatre using appreciative inquiry. According to Trajkovski et al. (2013), appreciative inquiry is more concerned with building on the strengths and opportunities of the phenomenon studied, rather than focusing on the problems. It emphasises offering a flexible opportunity to facilitate change from the grassroots up. However, the appreciative inquiry does not ignore the existence of problems. The 4-dimensional (4-D) relational cycle of appreciative inquiry was adopted in this article (see Figure 1).

The phases were delved into in this section (Table 1).

**TABLE 1:** Appreciative inquiry phases and description.

Appreciative inquiry phase	Description of how the phase was applied
Discovery phase	Participants’ experiences and expressions of innovative ways of communicating educational health messages through theatre were explored.
Dream phase	Participants’ aspirations regarding communication of educational health messages through theatre were explored.
Design phase	Participants’ views about the methods and processes that can be used to realise their aspirations regarding communication of educational health messages through theatre were explored.
Destiny phase	Participants’ views regarding the measures that should be in place to sustain communication of educational health messages were explored.

## Research methods

### Study setting

The study was conducted in Limpopo province, South Africa. Limpopo is one of the nine provinces in South Africa which is in the far north of South Africa, bordering countries like Mozambique and Zimbabwe. The province is predominantly rural, with a fair number of villages still running under the Kings and Queens administration. As a result, cultural and the Indigenous practices such as *Merumba* (drums), *Tshigombela* (girls group song), et cetera, are common, and used to convey valuable information to the communities, including educational health information.

### Population

The population in this study comprised an Ubuntu theatre group from a rural province in South Africa. The group comprises 30 important professionals in transdisciplinary healthcare services and youth development and is popular for singing, dancing and using theatre to communicate educational health messages from a traditional and Ubuntu perspective. As a result, the population size was computed as 30.

### Sampling

A non-probability purposive sampling method was used. According to Polit and Beck (2022), a non-probability purposive sampling approach is used when the researcher purposefully includes participants who have the potential to provide rich data. Similarly, this study used purposive sampling to ensure that the participants included in the appreciative inquiry would provide rich data about innovation in communicating educational health messages through theatre. A total sample size of 13 participants was used.

### Recruitment of participants

The researcher contacted the Ubuntu community model in nursing project’s primary investigator to request access to the Ubuntu Theatre group leader. The primary investigator is the custodian of the Ubuntu Theatre group because it falls within the larger Ubuntu community model in nursing project. This makes the primary investigator of the project the gatekeeper. Thereafter, a meeting for the recruitment of potential participants was arranged with assistance from the mediator. The mediator facilitated the process of arranging the initial recruitment meeting by linking participants with the researcher. After the initial recruitment meeting, an appointment was set to meet the group to share information about the study and secure informed consent from each participant. After explaining its contents, participants could read the information sheet and consent form independently. Informed consent for participation was then ensured. Verbal consent was also obtained from participants before data collection to affirm the consent.

### Data collection

Data were collected by two moderators from the two focus group discussions through conference calls. The two moderators were the first and second authors of this manuscript. The focus group guide was translated by a language expert into XiTsonga to allow maximum expression about the phenomena by participants. The four question items, as described by the 4-D of appreciative inquiry, were used to guide the focus group discussion. The participants were asked to describe their best experience of using theatre to communicate educational health messages from an Ubuntu perspective, to describe their ideal image of using theatre to communicate educational health messages in an Ubuntu perspective, to describe the processes that should be in place to support the use of theatre to communicate educational health messages from an Ubuntu perspective and to describe the measures that should be in place to sustain the use of theatre to communicate educational health messages from Ubuntu’s perspective. No identifying information was collected to ensure anonymity and confidentiality of the participants. Permission was sought from participants to record the conference call using an audio recorder feature for conference calling. In addition, the second moderator wrote notes for triangulation purposes and follow-up questions.

### Data analysis

Deductive thematic content data analysis was used. The authors used a deductive thematic content data analysis method because the study followed an appreciative inquiry research design. Therefore, the main aim was to describe the 4-Ds of appreciative inquiry within the collected data. Data analysis was conducted independently by the two authors in this study, who are established researchers locally and internationally. After analysing data independently, both authors met to discuss the findings and to agree with the themes and sub-themes. Creswell and Creswell’s (2023) steps of data analysis, namely organising and preparing data for analysis, reading all the data, coding all the data, generating themes, and representing the themes, were used.

### Measures to ensure trustworthiness

Lincon and Guba’s (1985) general criteria for ensuring trustworthiness in qualitative research which is credibility, transferability, dependability and confirmability were used. To elaborate, the authors used triangulation methods to ensure credibility. Transferability was ensured by detailed research methods to ensure that the findings could be applied to the next population. Dependability was ensured by working with two experienced researchers who analysed and coded data. Lastly, confirmability was ensured by following the research methods expressed in this manuscript.

### Ethical considerations

The study is part of a larger Ubuntu Community Model in Nursing project (NRF Project number 120441). As part of the bigger project, the study received ethical approval from the University of Pretoria’s Faculty of Health Sciences Research Ethics Committee (Ref number: 192/2020- Line 4). Furthermore, the study used the guidelines for conducting studies with human participants as stipulated in the 1969 Helsinki Declaration and South Africa’s 2015 Department of Health’s Ethics in Health Research Guidelines.

## Results

The findings are described further in the text, guided by the 4-Ds of appreciative inquiry design: discovery, dream, design, and destiny phases, including the demographic characteristics of the participants.

### Demographic data

The demographic characteristics of participants are described in Table 2.

**TABLE 2:** Demographic characteristics of participants.

Participant	Gender	Age (years)	Occupation
**Focus Group 1**			
1	Female	51	Employed
2	Male	20	Unemployed
3	Male	24	Unemployed
4	Female	22	Student
5	Female	24	Student
6	Female	64	Unemployed
**Focus Group 2**			
1	Male	24	Student
2	Male	27	Student
3	Male	22	Student
4	Female	65	Unemployed
5	Male	24	Employed
6	Male	30	Employed
7	Female	28	Unemployed

Participants’ ages ranged from 22 years to 65 years and above, with the majority (*n* = 7) being males. A total of four participants were still students, with only two of the participants on retirement. As a result, these demographics embodied the characteristics of intergenerational dialogues using theatre. Only two participants were employed, suggesting that most (*n* = 11) were fully invested in the theatre group. The themes and sub-themes are presented as discussed further in the text.

### Discovery phase

The discovery phase explored the participants’ expressions of the innovative ways to communicate educational health messages through theatre. Participants were asked to describe their best experience of using theatre to communicate educational health messages. Four themes emerged, namely: breaking language and cultural behaviours, playful pedagogy, using local language to communicate educational health care messages, enhancing memory through art, and community engagement in the form of relaying messages. The results are discussed further in the text.

### Breaking language and cultural barriers

According to participants, theatre has enabled them to break the barriers of culture and language in communicating educational health messages. Participants explained how much they use local language to convey their messages using theatre. The importance of knowing the local language, accompanied by cultural relevance, was highlighted by participants as being of utmost importance in their effort to convey health-related messages to the audiences. The participants indicated how culturally unacceptable language and behaviour can be more damaging than helpful. The importance of culturally congruent theatre was overly emphasised. One participant said:

‘[*M*]aam!!! Knowing the local language and culture is an advantage when giving information to local people about health issues. We used acceptable language and acted within the acceptable standards concerning their culture to communicate health messages. You could see that the participants were excited, and they participated. They felt respected and could identify with some information, which enhanced learning.’ (Participant 2, FDG 1, Male, 20 years old)

### Theatre is a playful pedagogy

Theatre was said to be a playful pedagogy described as the best medium for presenting the transmitted emotional, stressful situation in a lighter mood. Theatre was said to have been used to describe the stressful situation that people go through when suffering from debilitating diseases. This was a useful way of developing empathy among the participating audiences. This is what one of the participants said:

‘There is a high level of stigma and discrimination that people living with certain conditions endure in their everyday life, so in this case, it was necessary to highlight their everyday plight to change the mindset of the audiences, especially in the rural areas.’ (Participant 4, FDG 1, Female, 22 years old)

The other participant said:

‘You see, one thing that I love about using theatre or should I say arts to communicate health messages, is ehh, you know its ability to incorporate the artistic features to euphemise the even complex message. Take drama for example….’ (Participant 2, FDG 2, Male, 27 years old)

### Enhancing memory through art

Theatre as a teaching pedagogy was the best tool for enhancing memory. It was said that more often, participants have observed how audiences imitate what they have been doing or ask the theatre group to teach them songs, for example. The participants indicated that this reaction confirms that learning has occurred, and the audience might continue enacting or singing about what they have been taught. One participant said:

‘[*I*]t is always humbling to see some of the audience trying to imitate what you were doing or singing about the message you conveyed to them. For example, they continued to sing the song about ubuntu caring values and principles.’ (Participant 5, FGD 1, Female, 24 years old)

The other participant added by saying:

‘Indeed! And that’s the beauty of using theatre to communicate educational health messages. I mean, a song or a rhythm are very easy to remember, so we take advantage of that to ensure that, what the audience remember about a specific scene or act should be educational in nature. Especially the youth.’ (Participant 3, FDG 1, Male, 24 years old)

### Community engagement in the form of relaying messages

Community engagement as a way of conveying health messages was said to be one of the best innovative methods. Participants described how they work with communities to co-create health-related information and identify the best theatre medium to convey such a message. This was exciting as the community developed a sense of ownership that may increase adherence, which can be in the form of changing the lifestyle. Participants said:

‘It is always exciting when community members participate in creating health-related drama or songs. they usually identify the lyrics if it’s a song and the accompanying dance. they always own the product, improving our collaboration based on Ubuntu’s values and principles.’ (Participant 3, FGD 2, Male, 22 years old)

### Dream phase

As it is named, the dream phase is about envisioning the aspirations that could be brought about in the affirmative topic of choice (Trajkovski et al. 2013). As a result, five themes emerged: decolonising the health education system, developing educational programmes for actors to authenticate educational messages, expanding educational platforms, professionalising the actors, and formalising theatre as a teaching strategy. The themes are discussed in this section.

### Decolonising the health education system

The participants described using Ubuntu values and principles when communicating health messages to the audience. They described how these values and principles are infused in dramatising different situations or health information to the audiences. The use of Ubuntu-infused theatre also allows them to bring up culturally congruent messages, which is an area that was ignored as a teaching methodology. The blending of indigenous means of conveying messages, even research results during conferences, was said to be an innovative way of decolonising the health education system dominated by Western pedagogies foreign to the African people. The highlight of the decolonisation of the health education system was said to be when the theatre was infused to communicate research results during the Ubuntu conference. This is what some of the participants said:

‘When we use theatre to communicate health messages to different age groups, we normally align our theatre be it songs, dance or poetry, to the culturally acceptable practices to ensure that our health messages are well received and have lasting impact.’ (Participant 2, FGD 1, Male, 20 years old)

Another participant said:

‘You know, I’m not a professor however I managed to infuse Ubuntu in the way in which I communicate the results of the study that was conducted on African indigenous people. I think I managed to bring life to the results by bringing the accompanying emotions to the results, enacting the feelings underlying the results.’ (Participant 4, FDG 2, Female, 65 years old)

### Educational programme for capacitating actors to authenticate educational health messages

Participants felt it was essential to be continuously capacitated with authentic health messages to ensure their audiences always receive updated health information. This was said to be very important and was said to be achieved by having a functional collaboration with health personnel, including academics. It was also suggested that regular workshops and the supply of updated pamphlets to the group could be beneficial. Participants also indicated their need for a creative arts person to teach them more theatre skills. One participant said:

‘We need regular workshops on health-related matters to ensure that we have correct health information that is also updated.’ (Participant 1, FDG 2, Female, 51 years old)

Another participant said:

‘Regular workshops will capacitate us and can open more doors for us.’ (Participant 6, FDG 2, Male, 30 years old)

### Expanding educational platforms (e.g. social media, TV, radio)

Expanding the educational platform was said to be something that participants aspire to do in the future. Participants indicated that they would want to venture into radio, for example, where they would develop health-related stories that they could enact on radio and television to reach a bigger audience. It was also said to be essential to use social platforms like TikTok and short stories as adverts and videos that people, especially youth, can continuously watch on their own. Expanding educational platforms was said to be what they aspired to do to reach bigger audiences, unlike now, where they rely on physical appearance. Participants said:

‘We want to venture into more prominent educational platforms like TV and radio. [*Laugh*] This can expose us and increase our audience’s number.’ (Participant 3, FDG 1, Male, 24 years old)

The other participant said:

‘Well, for me I also think that expanding our footprints in social media platforms should be something to consider to reach a broader audience, especially the youth. I mean, we cannot shy away from the fact that a lot of them spend a considerable amount of time in platforms like Facebook and recently TikTok. So, capitalising on that can really assist to preach the Ubuntu innovation in communicating educational health messages.’ (Participant 3, FDG 1, Male, 24 years old)

### Professionalising the actors or participants

The participants expressed the need to professionalise their theatre careers because, at the moment, they were using their skills without having gone through training. The participants felt that if they could be offered some form of training, the training could open opportunities that could eventually make them join the formal job market. Professionalising could improve their theatre skills and health knowledge to equip their audiences better. One participant said,

‘I see myself as a professional theatre artist in the near future. However, I feel that it can be possible if I undergo training to become confident with what I do and collaborate with health professionals to ensure that health messages are correct.’ (Participant 5, FDG 2, Male, 24 years old)

### Formalising theatre as a teaching method

There was a feeling among participants that as theatre enhances memory, it should be used as a teaching method. Participants felt that theatre as a teaching method could be easily used in health-related programmes. They felt this could be achieved by collaborating with established theatre schools and curriculum development specialists. One of the participants said:

‘I think that theatre can be adopted and used in formal education as a recognized teaching method. This will mean the nursing and other health lecturers will be trained to use theatre as a teaching strategy.’ (Participant 6, FDG 2, Male, 30 years old)

### Design phase

The Design phase is described by Cooperrider, Whitney and Stavros (2008) as a phase wherein the idea of ‘what should be’ is constructed. In other words, the researchers should answer the question, ‘How to empower, learn, and adjust’ (Trajkovski et al. 2013). Three themes emerged and are discussed next.

### Development of a training programme

Participants felt it was essential to develop Ubuntu health course manuals that can be continuously updated with current information to keep participants abreast with new developments. Participants indicated that new conditions should also be added to the training programme when the disease profile changes. This is to ensure that they communicate authentic educational messages through theatre. They emphasised that the Ubuntu-infused content of the health conditions should also be added with references if there is a need for further reading. It was also indicated that different training on health-related conditions should be prioritised, with specific care taken regarding the local language and cultural practices. Collaboration was said to be significant among leaders of the stakeholders. Participants said:

‘Maam!!!! there is a need to restructure how we do our work. We need knowledge so that what we teach is not questionable and we are also confident about the health messages we deliver.’ (Participant 3, FDG 1, Male, 24 years old)

Another one said:

‘We need a structured training program that can give us grounding. You know, including materials like health course manuals because as much as we are artists, we need to be relevant with regard to the health-related content that we share with audience.’ (Participant, FDG 1, Female, 51 years old)

### Theatre used as an alternative playful pedagogy

Participants felt that theatre can be used as a playful pedagogy where short videos can be developed and used as automated health information sharing sessions in the waiting areas of the health care facilities. Including Ubuntu-infused messages in social platforms like *TikTok, Facebook* and *Instagram* was also suggested. These platforms could reach the younger generation, especially the youth. Participants suggested they could also be used as standardised patients during practical teaching and examination:

‘I mean, looking at the current generation, I think platforms such as TikTok can work to our benefit. Rather than just scrolling for leisure, they can be used for information sharing.’ (Participant 2, FDG 2, Male, 27 years old)

### Collaboration with key stakeholders

The participants explained that collaboration with key stakeholders could be the driver of the use of theatre to communicate educational health messages. This is because, they expressed that they understand that stakeholders such as institutions of higher learning, the local art department, and the Department of Health must collaborate to realise this dream. This is how they expressed the issue of collaboration:

‘I mean, this thing should be err. .err a joint effort. For example, the Department of Art and Culture can assist in shaping our craft while universities capacitate us with health-related knowledge, and consumers are within the health department. I don’t know if you understand me.’ (Participant 6, FDG 1, Female, 64 years old)

### Destiny phase

Destiny is the final phase of the appreciative inquiry concerned with exploring measures to sustain the envisioned future (Cooperrider et al. 2008). Four themes emerged and are discussed in this section.

#### Teaching life skills

Teaching life skills to participants was one of the requirements that participants felt was necessary. This was one of the strategies that they recommended as a way in which the sustainability of the project could be ensured. They emphasised the importance of having life skills programmes to assist them in navigating life as most of them are still young. Life skills were said to be a vehicle that can help them set goals and deal with different social and emotional issues that confront them. According to participants, life skills can maintain group cohesion. One participant said:

‘You know, Maam!!! [*laugh*], As a youth, we are confronted with different problems. I mean social and emotional issues. We should have skills for dealing with these issues.’ (Participant 3, FDG 1, Male, 24 years old)

Another one said:

‘In addition to what participant 3 said, I think having such skills will go a long way in ensuring the longevity, if that’s the correct word [*laughs*], of the group because it gets tough sometimes.’ (Participant 5, FDG 1, Female, 24 years old)

#### Expanding the Ubuntu Theatre movement group

Expansion of the theatre group by opening the group to new participants was suggested. It was felt that the group could be enlarged to a reasonable number so that when some members leave, the void is not that big. It was said to be a succession plan for the theatre group that would ensure it did not collapse. Furthermore, participants suggested that training of the trainers was essential as this may give rise to different theatre groups in different places to ensure that the intended health messages reach a broader population in different areas. Participants felt it would be easier for the project leaders to manage the different groups of trainers if they had trainers in different areas. This was also said to be a cost-containment strategy, as project leaders must be present in all pieces of training. One participant said:

‘I think there should be a program where trainers are trained to facilitate the ongoing group training. It is a capacity building and cost-containment measure.’ (Participant 1, FDG 1, Female, 51 years old)

Another participant said:

‘It is very important to ensure sustainability of the theatre group. By adding members, we can ensure that when people leave the group, there are people who will remain doing the job. This is an amazing job, however, if one gets employment, we can leave especially when the job opportunity is far away.’ (Participant 4, FDG 1, Female, 22 years old)

### Continuous review of the manuals and in-service training

Participants felt that they needed to have current health information to not convey outdated messages. This was achieved by continuously updating information in the manuals and holding regular workshops on theatre skills refinement. Including local indigenous experts was also suggested to ensure that content and role plays or demonstrations are culture sensitive. One participant said:

‘Health is a dynamic field with constant changes in disease profiles and so on. The discoveries of diseases and their management are not static. It is therefore important to always have updated information to ensure we give correct information.’ (Participant 1, FDG 2, Male, 24 years old)

### Sustaining collaborative partnerships

Participants felt it was essential to sustain the theatre group, including a good marketing strategy, making good networks, and funding to run the project. Participants said it was essential to market their actions to get more events and generate funds. They also felt that it is necessary to be capacitated on how to raise funds by writing funding proposals to relevant organisations. Collaboration with relevant organisations was also said to be essential. They emphasised that such collaborative partnership should include the health department, communities and community leaders, as the custodians of health consumers. The participants also felt that as part of their sustainability strategy, the theatre group needed to document their success stories. This was said to be a strategy that could help make the group known or even attract funding from different organisations. As a result, success stories of the group on how it uses theatre to communicate educational health messages can be documented and copied even in future, or by emerging groups from other areas. One participant said:

‘You know, networking with relevant organisations to market us and connect us to relevant events is essential for sustainability. But funding remains an issue of concern because sometimes we get the gigs, but logistics surrounding how to get there, you know, accommodation sometimes, that now becomes a problem.’ (Participant 4, FDG 1, Female, 24 years old)

Another participant said:

‘I think we need to write down our success stories so that when people want to know about us, we can go into our archives to retrieve this information.’ (Participant 5, FDG 1, Female, 24 years old)

## Discussion

Theatre as a formal educational pedagogy goes a long way in the provision of educational health information. Using theatre to communicate educational health messages has the ability to break language and cultural barriers imposed by the contemporary education system. This is achieved through its features which go beyond audio and visual performances, but inclusive of artistic features such as rhythmic clapping, gestures, and drawing. This helps in reaching various audiences with varied disabilities like the deaf and dumb, including breaking intercultural barriers. A study conducted by Jones (2020) affirms that theatre is an ancient teaching method used to sustain intergenerational dialogues of educational health messages. Furthermore, theatre as an educational tool has the potential to decolonise the current education system by infusing the Afro-centric approach (Carr & Hooker 2023; De Souza 2022; eds. Lupton & Leahy 2021). Thus, theatre as a pedagogy has been successfully used to breach the language and cultural gap between generations when communicating educational health messages (De Souza 2022).

Despite the success stories of theatre as an alternative pedagogy, it has not been formalised as a formal teaching strategy at institutions of higher learning. Instead, theatre as an alternative pedagogy is relegated to Indigenous knowledge systems. This means that there is still a handful that needs to be done to get to a point wherein theatre is recognised as an alternative teaching strategy. Some of the suggestions to recognise theatre as an alternative pedagogy include incorporating theatre within the health care sciences curriculum. However, the discourses that exist around the incorporation of theatre within the mainstream curriculum include whether it will be feasible to balance between entertainment, accuracy of the scientific information, and humour (Sharma et al. 2021). For example, while some content can be acted out to maximise learning, instead of being documented, caution must be taken to ensure that the core content of the health message is delivered and not clouded by entertainment and humour. According to a study conducted by Stoneham and Feltham (2009), one of the advantages of theatre is its ability to maximise learning through its playful pedagogical approaches especially when used in clinical simulation. Furthermore, the use of drama in communicating health messages offers a unique approach to health education in low-literate rural communities (Asante 2016). In the same breath, there is a proposition to expand platforms for theatre to communicate educational health messages; these would even include social media platforms.

The authors argue that, as an ancient teaching strategy, it cannot be disputed that there are numerous missed opportunities from its inception in terms of sustaining theatre as an educational tool to communicate health messages. These therefore include strategies such as appreciating and documenting the success stories of theatre and are recommended as an alternative pedagogy. Similar findings are echoed by a study conducted by Sharma et al. (2021) which states that there is poor documentation of the effectiveness of entertainment-educative approaches to address public health concerns. Apart from documenting success stories of theatre as an educational strategy, there is a need to establish and sustain collaborative partnerships between various departments that play a role in professionalising the actors. This is to ensure authenticity of the educational health messages and develop role players as professional actors. By doing so, it will be seaming less to adopt theatre as an alternative pedagogy to communicate educational health messages. This is supported by French, Mulhern and Ginsberg (2019) who cite that using theatre as an educational tool has a potential to drive opportunities for social learning and collaboration. Furthermore, Hobson et al. (2019) cite that theatre is regarded as a teaching method of choice when faced with difficult-to-teach subjects. This involves having to teach about complex health issues such as sex dialogues between the young and the old within predominantly traditional societies.

### Limitations

The study used conference calls as a method to collect data from participants because it was difficult to get more participants in one setting during data collection. This method is seen as a limitation because it does not give the opportunity for the moderators to read non-verbal cues that are essential in corroborating data obtained from participants.

### Recommendations

The recommendations were made to the nursing education, nursing practice and nursing research as discussed next.

#### Nursing education

The authors recommended that the theatre should be adopted as an alternative and formal educational pedagogy within health sciences curriculum. This means that as theatre pedagogy is an indigenous pedagogy, it is recommended that nursing education institutions benchmark on the best practices of theatre to incorporate that within the contemporary education systems. This helps in infusing Ubuntu and the Afri-centric approach in communicating educational health messages.

#### Nursing practice

It is recommended that the clinical practice environments adopt theatre as an alternative approach to communicate educational health messages. This is because, although theatre is a playful pedagogy, it maximises learning and messages communicated through theatre are memorable unlike the generic health education. Furthermore, theatre uses local language and is best suited to communicate complex and controversial health messages.

#### Nursing research

The authors recommend that success stories of theatre as an educational and alternative health pedagogy should be documented. As a result, it is recommended that more research should be conducted in similar topic areas to explore even further how theatre can be used to communicate educational health messages effectively.

## Conclusion

Theatre has proved to be an innovative teaching strategy for decades, including in today’s health education system. However, the method is not widely used as there are no formal assessments tools for it. By using Ubuntu innovation to communicate educational health messages through theatre, there can be maximised learning. This is because, theatre is a playful pedagogy and is best suited to communicate complex health issues. Also, infusing Ubuntu philosophy in communicating would greatly assist in breaking the cultural and language barriers since Ubuntu philosophy embraces communalism.
